# Association between PCSK9 inhibitors and acute kidney injury: a pharmacovigilance study

**DOI:** 10.3389/fphar.2024.1353848

**Published:** 2024-08-01

**Authors:** Hailing Liu

**Affiliations:** Hunan Provincial People’s Hospital, The First Affiliated Hospital of Hunan Normal University, Changsha, China

**Keywords:** PCSK9 inhibitors, acute kidney injury, evolocumabEvolocumab, aAlirocumab, pharmacovigilance

## Abstract

**Background:**

PCSK9 inhibitors are a novel class of lipid-lowering medications, and numerous clinical studies have confirmed their significant role in improving the progression of chronic kidney disease. However, recent case reports have indicated new evidence regarding their association with acute kidney injury (AKI), with some patients experiencing acute tubular injury after PCSK9 inhibitors use.

**Objectives:**

To clarify the relationship between PCSK9 inhibitors and AKI, we conducted a pharmacovigilance study.

**Methods:**

Using the Food and Drug Administration Adverse Event Reporting System (FAERS) database from the third quarter of 2015 to the fourth quarter of 2022, a disproportionality analysis was employed to identify adverse events suggestive of AKI after PCSK9 inhibitors use. The drugs of interest included evolocumab and alirocumab.

**Results:**

A total of 144,341 adverse event reports related to PCSK9 inhibitors were analyzed, among which 444 cases were suspected of AKI for evolocumab, and 172 cases for alirocumab. Evolocumab had a greater impact on AKI in males (ROR 1.4, 95% CI 1.54–1.69). The ROR and 95% CI for evolocumab and Alirocumab were 0.13 (0.12–0.14) and 0.26 (0.23–0.30) respectively. Further analysis of AKI associated with the concomitant use of PCSK9 inhibitors with cephalosporins, furosemide, torsemide, pantoprazole, omeprazole, and esomeprazole revealed ROR and 95% CI of 0.38 (0.23–0.62), 0.38 (0.31–0.48), 0.18 (0.08–0.38), 0.23 (0.17–0.29), 0.20 (0.16–0.26), and 0.14 (0.10–0.20) respectively.

**Conclusion:**

Through the FAERS database, we analyzed the clinical characteristics of AKI associated with PCSK9 inhibitors, exploring its risks. Our findings suggest that PCSK9 inhibitors might have a potential protective effect against AKI and exhibit similar effects when co-administered with other nephrotoxic drugs.

## Highlights

• PCSK9 inhibitors may be potentially protective against AKI to some extent and show similar effects when combined with other nephrotoxic drugs.

## 1 Background

Proprotein Convertase Subtilisin/kexin Type 9 (PCSK9) is a serine protease synthesized by liver cells, which circulates in the bloodstream and forms complexes with Low-Density Lipoprotein Receptors (LDL-R). These complexes are eventually degraded in lysosomes within liver cells, leading to decreased surface LDL-R levels. LDL-R is a crucial factor for liver cell uptake and metabolism of LDL cholesterol (LDL-C). Consequently, PCSK9 elevates LDL-C levels in the body (Rosenson et al., 2018). Research indicates that elevated serum LDL-C levels are an independent risk factor for Atherosclerotic Cardiovascular Disease (ASCVD). Clinical trial data have demonstrated a correlation between lowering LDL-C levels and reducing cardiovascular risk (Silverman et al., 2016). Therefore, reducing LDL-C is a key strategy for primary and secondary prevention of ASCVD (Stone et al., 2014). PCSK9 inhibitors significantly reduce LDL-C levels in the human body through two main pathways: first, by inhibiting the binding of PCSK9 to LDL-C receptors and second, by intervening in the synthesis and processing of PCSK9 (Lagace, 2014). In a Phase I clinical trial, the anti-PCSK9 siRNA ALN-PCS reduced free PCSK9 levels by 70% and lowered LDL-C levels by 40% (Fitzgerald et al., 2014).

PCSK9 in circulation primarily originates from the liver. Additionally, it is expressed in the pancreas, kidneys, intestines, and central nervous system. While PCSK9 regulates cholesterol metabolism by modulating LDL receptor expression in the liver, *in vitro* and *in vivo* studies suggest that PCSK9 is involved in various other physiological processes (Stoekenbroek et al., 2018). Studies (Toth et al., 2018) have demonstrated the lipid-lowering effects of PCSK9 inhibitors in patients with chronic kidney disease (CKD), showing good safety and efficacy. At week 24, LDL-C reduction ranged from 46.1% to 62.2% in patients with or without renal impairment, respectively. The overall incidence of adverse reactions was similar between the treatment and control groups (82.1% vs. 82.8% in CKD patients; 78.4% vs. 78.2% in non-CKD patients). Furthermore, the more severe the CKD, the greater the absolute reduction in cardiovascular deaths, myocardial infarctions, or strokes associated with PCSK9 inhibitors use (Charytan et al., 2019). Patients with CKD stage 3 or higher, when treated with PCSK9 inhibitors for 30 months, experienced a significantly greater absolute risk reduction compared to patients with normal renal function, with reductions of −2.5% (95% confidence interval (CI) −4.7% to −0.4%) and −1.7% (95% CI: -2.8%–0.5%) respectively. Studies (Haas et al., 2016; Jatem et al., 2021) also revealed that knockout mice with nephrotic syndrome lacking liver Pcsk9 exhibited a 40%–50% reduction in plasma cholesterol and triglycerides. Patients with primary refractory nephrotic syndrome showed an average LDL-C reduction of 36.8% ± 4.9% mmol/L after 4 weeks of PCSK9 inhibitors therapy, which remained stable throughout the follow-up period. In contrast, total cholesterol or LDL-C levels showed no significant change in the statin-treated group, suggesting that PCSK9 inhibitors might be effective and safe alternatives for treating hypercholesterolemia associated with refractory nephrotic syndrome. Recent research (Skeby et al., 2023) has reported interactions between PCSK9 and Megalin in proximal tubular cells. By affecting megalin-driven protein reabsorption, PCSK9 influences urinary protein excretion. PCSK9 inhibitors increase renal megalin in mice with kidney disease, simultaneously reducing urinary albumin excretion. This discovery provides new strategies for treating CKD.

However, there are also case reports (Jhaveri et al., 2017; Pickett et al., 2020) suggesting that PCSK9 inhibitors can induce AKI. One case involved a 62-year-old female patient (Jhaveri et al., 2017) with comorbidities such as heart disease, hyperlipidemia, Stage 5 CKD, and hypertension. The patient denied using any non-steroidal anti-inflammatory drugs, antibiotics, or herbal supplements. After using alirocumab, her serum creatinine increased from a baseline of 2.3 mg/dL to 5.0 mg/dL. Kidney biopsy revealed acute tubular injury and necrosis. The authors speculated that this might be due to the overexpression of renal PCSK9 during the inhibition of the inflammatory process, which is actually a protective response to cellular damage. Another case involved a 72-year-old male patient (Pickett et al., 2020) with coronary artery disease, statin intolerance, and Stage 3 CKD. After using alirocumab, the patient also experienced acute tubular injury detected in kidney biopsy. Upon discontinuation of the medication, serum creatinine levels returned to baseline.

Currently, there are no specific research reports on the correlation between PCSK9 inhibitors and AKI. Whether they can reduce the risk of AKI remains unknown. AKI is characterized by a rapid decline in kidney function, accompanied by the accumulation of metabolic products such as creatinine and urea, constituting a clinical syndrome (Bellomo et al., 2012). It is typically transient and often overlooked. However, AKI is associated with increased risk of mortality. A prospective study (Kaddourah et al., 2017) involving 4,683 patients from multiple countries showed that by day 28, 543 patients developed severe AKI, leading to an increased risk of death (adjusted odds ratio 1.77; 95% CI, 1.17–2.68). The SEA-AKI study (Kulvichit et al., 2022) also reported an in-hospital mortality rate of up to 14.6% for AKI. Moreover, as the severity of AKI increases, the risk of death also rises (Hoste and Kellum, 2006; Hoste et al., 2015). The adjusted odds ratios for in-hospital death in AKI stages 1, 2, and 3 were 1.679 (95% CI 0.890–3.169; *p* = 0.109), 2.945 (95% CI 1.382–6.276; *p* = 0.005), and 6.884 (95% CI 3.876–12.228; *p* < 0.001), respectively (Hoste et al., 2015).

To further elucidate the correlation between PCSK9 inhibitors and AKI, we conducted a pharmacovigilance study using FAERS database. This spontaneously reported adverse reaction database is freely accessible and includes a diverse population and various medications. It is useful for capturing adverse events occurring shortly after drug exposure and can detect adverse events not found in clinical trials, especially for rare events with low background rates.

## 2 Methods

### 2.1 Data source

The data for this study were obtained from FAERS database, a large, publicly accessible database consisting of adverse reaction cases reported by various populations, including healthcare professionals, consumers, and lawyers. AERSMine, a website developed based on the FAERS database, provides convenient and precise search services. It has become a mature tool for mining and analyzing drug adverse reactions (Sarangdhar et al., 2016; Xia et al., 2022; Xia et al., 2023). We conducted an observational, retrospective, cross-sectional pharmacovigilance study using post-marketing data from the FAERS database, spanning from the third quarter of 2015 to the fourth quarter of 2022. Ethical approval was not required for this study as it utilized de-identified data.

### 2.2 Drug selection and adverse reaction definition

The drugs of interest in this study were evolocumab and alirocumab, both approved by the FDA and EMA in 2015. We used the preferred terms (PTs) under the category of acute kidney failure based on the standard MedDRA query (SMQ) as keywords to identify target adverse reactions. Selected PTs are showed in Supplementary Material. For a better description of the characteristics and further analysis of PCSK9 inhibitors’ association with AKI, we specified the drug role as “primary suspect.”

### 2.3 Drug interaction analysis

In clinical practice, combination therapy is common. However, the impact of concomitant use of PCSK9 inhibitors with other medications on AKI is not well understood. Therefore, we analyzed commonly used nephrotoxic drugs to explore the effect of their co-administration with PCSK9 inhibitors on AKI. A nationwide cross-sectional survey (Liu et al., 2021) involving 23 academic hospitals in 17 provinces of China revealed that the top three categories of drugs causing drug-induced AKI were antimicrobial drugs, diuretics, and proton pump inhibitors (PPIs). Within these categories, the most common drugs were cephalosporins, glycopeptides, and carbapenems for antimicrobial drugs; furosemide, mannitol, and torsemide for diuretics; and pantoprazole, omeprazole, and esomeprazole for PPIs. We analyzed the occurrence of AKI when PCSK9 inhibitors were co-administered with these nine drugs.

### 2.4 Data mining and statistical methods

In this study, we employed the Reporting Odds Ratio (ROR) method, a disproportionality analysis, for risk analysis and mining. This method is also the most commonly used approach in current pharmacovigilance studies (Anand et al., 2019; Zhou et al., 2023). ROR with positive signal detection criteria was defined as having a report count ≥3 and a lower limit of the 95% CI of ROR >1. The method for calculating the ROR is provided in the Supplementary Material. Additionally, based on existing literature reports, we analyzed the risk of AKI when PCSK9 inhibitors were co-administered with common nephrotoxic drugs. Subgroup analyses were performed in different gender, age groups and underlying diseases to enhance the reliability and stability of the research results. Pearson’s chi-squared test or Fisher’s exact test was used to compare the reporting of PCSK9 inhibitors-related AKI, with statistical significance determined by a 95% CI, and *p* < 0.05 was considered significant. Statistical analysis was performed using SPSS 25.0 and Microsoft Excel 2019.

## 3 Results

### 3.1 Basic characteristics

**FIGURE 1:**
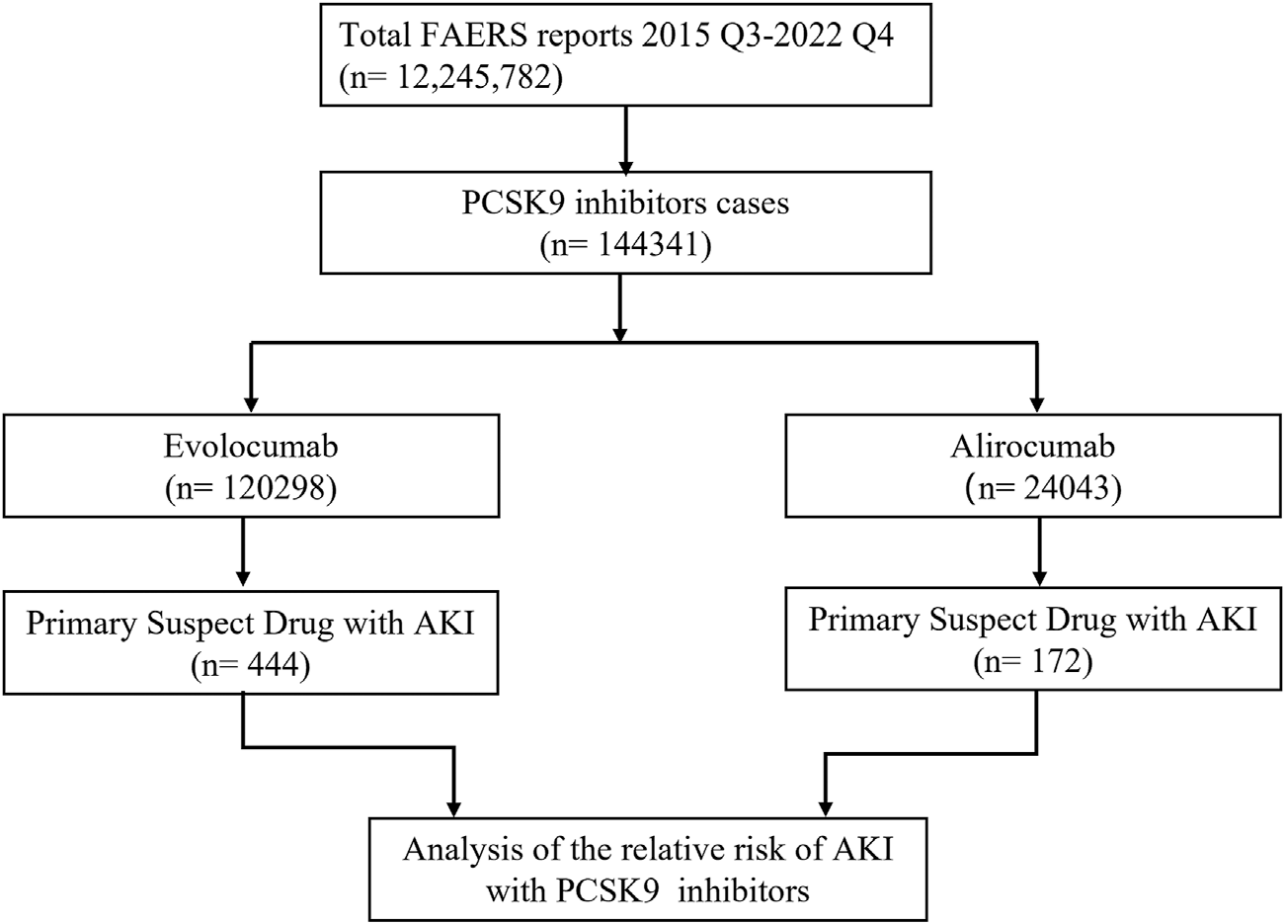
Flow chart of study design. In this study, 12, 245, 782 reports were retrieved from the FAERS database during the third quarter of 2015 to the fourth quarter of 2022. Among these reports, 144,341 cases were linked to adverse reactions related to PCSK9 inhibitors. To investigate the correlation between PCSK9 inhibitors and AKI, we selected the primary suspect drug for further analysis.

**FIGURE 2:**
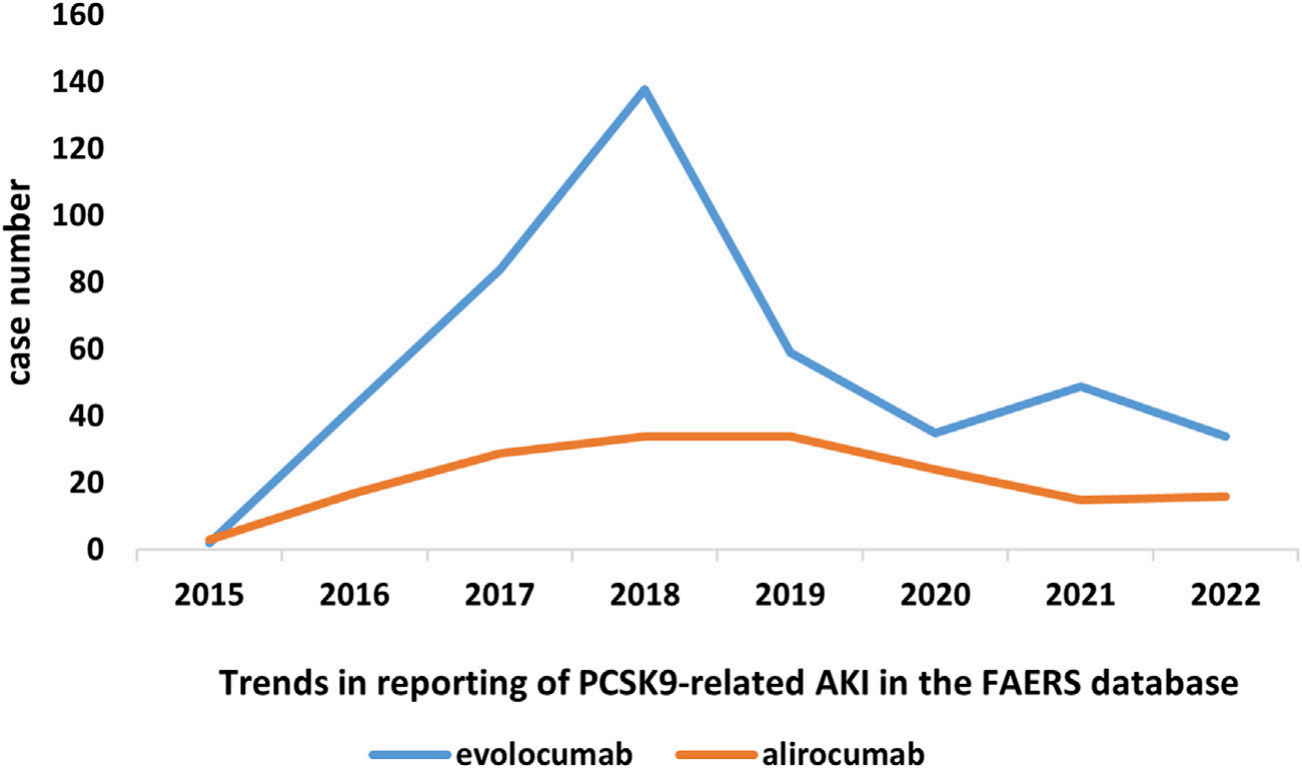
Trends in reporting of PCSK9 inhibitors-associated AKI in the FAERS database.

In this study, we ultimately included 444 cases of evolocumab and 172 cases of alirocumab, as shown in Figure 1. All of which were primary suspect cases associated with AKI, reported in the FAERS database from the third quarter of 2015 to the fourth quarter of 2022. Table 1 summarizes the clinical characteristics of these cases. The reporting proportions of evolocumab-related AKI were similar between males (48.6%) and females (46.4%), *p* = 0.55, while alirocumab-related AKI was notably higher in females, *p* < 0.05. Evolocumab-related AKI cases were primarily reported in individuals aged 65 and above, with the rest distributed mainly between 25–65 years and in age-unreported cases. In contrast, alirocumab-related AKI cases were relatively evenly distributed among individuals aged 25–65, 65 and above, and those with unknown ages, accounting for 27.9%, 34.3%, and 37.8%, respectively. Notably, AKI related to both drugs almost did not occur in individuals below 24 years old, likely due to the specific demographic of users. Evolocumab-related cases were predominantly reported by healthcare professionals, while the pattern was reversed for alirocumab, *p* < 0.05. The number of reported AKI adverse reactions for both drugs gradually increased after market introduction, reaching a peak in 2018 and subsequently declining, as shown in Table 1 and Figure 2. Specifically, evolocumab-related AKI cases reached 138 in 2018, accounting for 31.1% of the total cases. The most frequently reported PTs for evolocumab and alirocumab-related AKI were “blood creatinine increased,” “renal failure,” “renal impairment,” and “acute kidney injury,” as listed in Table 2. The patient outcomes for AKI cases associated with evolocumab and alirocumab are presented in the Supplementary Material. Overall, the number of hospitalizations and other serious outcomes is higher, while instances of death, disability, and life-threatening conditions are relatively fewer.

**TABLE 1:** Characteristics of patients with PCSK9 inhibitors-associated AKI in FAERS database.

	Evolocumab N. (%)	Alirocumab N. (%)	*p*-Value
all AEs	120,298	24,043	
primary suspect drug with AKI	444 (0.37)	172 (0.72)	<0.05
gender			
males	216 (48.6)	49 (28.5)	<0.05
females	206 (46.4)	84 (48.8)	0.65
not reported	22 (5.0)	39 (22.7)	<0.05
age groups			
0–14	0 (0.0)	0 (0.0)	-
15–24	2 (0.4)	0 (0.0)	-
25–65	117 (26.4)	48 (27.9)	0.77
≥65	223 (50.2)	59 (34.3)	<0.05
not reported	102 (23.0)	65 (37.8)	<0.05
Reporter occupation			
healthcare professionals	324 (73.0)	68 (39.5)	<0.05
others	120 (27.0)	104 (60.5)	<0.05
Year			
2015	2 (0.5)	3 (1.7)	0.27
2016	43 (9.7)	17 (9.9)	0.94
2017	84 (18.9)	29 (16.9)	0.63
2018	138 (31.1)	34 (19.8)	<0.05
2019	59 (13.3)	34 (19.8)	0.06
2020	35 (7.9)	24 (13.9)	<0.05
2021	49 (11.0)	15 (8.7)	0.49
2022	34 (7.6)	16 (9.3)	0.61

Abbreviations: AEs, Adverse Events.

**TABLE 2:** Preferred terms reporting status for PCSK9 inhibitors-associated AKI.

Evolocumab		Alirocumab	
PTs	Case number	PTs	Case number
renal impairment	114	blood creatinine increased	44
renal failure	97	renal impairment	37
blood creatinine increased	68	renal failure	36
acute kidney injury	48	acute kidney injury	23
dialysis	29	blood urea increased	11
glomerular filtration rate decreased	28	glomerular filtration rate decreased	8
blood urea increased	24	proteinuria	8
renal function test abnormal	20	renal function test abnormal	6
protein urine present	10	renal tubular necrosis	6
urine output decreased	8	anuria	5
proteinuria	7	urine output decreased	5
renal transplant	6	dialysis	5
glomerular filtration rate abnormal	5	renal tubular injury	3
blood creatinine abnormal	4	renal transplant	3
peritoneal dialysis	4	oliguria	3

Abbreviations: PTs, Preferred terms.

### 3.2 Disproportionality analysis

In this study, we used ROR algorithm in disproportionality analysis to detect the association between AKI and the use of evolocumab and alirocumab. The results are presented in Table 3. We found a negative correlation between the use of these two drugs and the reporting of AKI, indicating a potential protective effect against AKI. Furthermore, evolocumab demonstrated a stronger protective effect compared to alirocumab, with ROR and 95% CI for evolocumab-related and alirocumab-related AKI being 0.13 (0.12–0.14) and 0.26 (0.23–0.30), respectively.

**TABLE 3:** Disproportionality analysis.

Drug	Primary suspect drug with AKI	ROR (95% CI)
evolocumab	444	0.13 (0.12–0.14)
alirocumab	172	0.26 (0.23–0.30)

Abbreviations: CI, confidence interval; ROR, reporting odds ratio.

### 3.3 Interaction analysis

In our analysis, we investigated the occurrence of AKI when PCSK9 inhibitors were combined with nine clinically common nephrotoxic drugs previously reported in studies (Liu et al., 2021). The results demonstrated that PCSK9 inhibitors could mitigate the nephrotoxic effects of cephalosporins, furosemide, torsemide, pantoprazole, omeprazole, and esomeprazole, with significant differences observed (ROR and 95% CI: 0.38 [0.23–0.62], 0.38 [0.31–0.48], 0.18 [0.08–0.38], 0.23 [0.17–0.29], 0.20 [0.16–0.26], and 0.14 [0.10–0.20], respectively). The results are presented in Table 4. However, conclusive results could not be drawn for the other three drugs due to insufficient case numbers.

**TABLE 4:** Interactions between PCSK9 inhibitors and common nephrotoxic drugs.

Drug A	Drug B	Patients total	NO. AKI	Proportion of AKI (%)	ROR (95% CI)	*p*-Value
cephalosporins	with PCSK9 inhibitors	371	17	4.58	0.38 (0.23–0.62)	<0.0001
	without PCSK9 inhibitors	121,428	13,568	11.17	1 (reference)	
glycopeptide	with PCSK9 inhibitors	29	3	10.34	0.57 (0.17–1.87)	0.46
	without PCSK9 inhibitors	52,346	8,856	16.92	1 (reference)	
carbopenems	with PCSK9 inhibitors	4	1	25.00	2.93 (0.30–28.17)	0.35
	without PCSK9 inhibitors	35,381	3,615	10.22	1 (reference)	
furosemide	with PCSK9 inhibitors	1,512	78	5.16	0.38 (0.31–0.48)	<0.0001
	without PCSK9 inhibitors	309,774	38,468	12.42	1 (reference)	
mannitol	with PCSK9 inhibitors	3	0	0.00	-	-
	without PCSK9 inhibitors	4,769	563	11.81		
torsemide	with PCSK9 inhibitors	223	7	3.14	0.18 (0.08–0.38)	<0.0001
	without PCSK9 inhibitors	42,018	6,509	15.49	1 (reference)	
pantoprazole	with PCSK9 inhibitors	1,543	63	4.08	0.23 (0.17–0.29)	<0.0001
	without PCSK9 inhibitors	309,700	49,232	15.90	1 (reference)	
omeprazole	with PCSK9 inhibitors	1933	68	3.52	0.20 (0.16–0.26)	<0.0001
	without PCSK9 inhibitors	373,769	56,482	15.11	1 (reference)	
esomeprazole	with PCSK9 inhibitors	606	29	4.79	0.14 (0.10–0.20)	<0.0001
	without PCSK9 inhibitors	188,105	49,652	26.40	1 (reference)	

### 3.4 Subgroup analysis

Table 5 and Table 6 presents the results of subgroup analysis. In patients with underlying disease, PCSK9 inhibitors still showed potential AKI protection, see Table 5. Evolocumab-related AKI was more likely to occur in males (ROR = 1.40, 95% CI = 1.54–1.69). In comparison with the population aged 65 and above, individuals between the ages of 25–65 appeared to have a slightly lower risk of AKI after using evolocumab, although this difference was not statistically significant. On the other hand, alirocumab showed a higher risk of AKI in patients aged 25–65, but again, this difference was not statistically significant. It is important to note that due to the limited number of reported cases in patients under 25 years old, a direct comparison for this age group could not be made. Additionally, the differences observed in this analysis require further investigation with larger sample sizes and more comprehensive prospective studies to draw definitive conclusions.

**TABLE 5:** PCSK9 inhibitors-associated AKI in patients with several underlying diseases.

Drug	Underlying diseases	NO. AKI	ROR (95% CI)
evolocumab	diabetes mellitus	5	0.11 (0.05–0.27)
	hypertension	11	0.15 (0.08–0.28)
	hyperlipidaemia	55	0.05 (0.04–0.07)
	heart failures	3	0.27 (0.09–0.85)
alirocumab	diabetes mellitus	1	0.10 (0.01–0.71)
	hypertension	6	0.13 (0.06–0.29)
	hyperlipidaemia	28	0.09 (0.06–0.13)
	heart failures	1	0.23 (0.03–1.72)

The number of adverse events reported with alirocumab in patients with diabetes and heart failure was less than 3 cases of AKI, and therefore does not pose a risk.

**TABLE 6:** PCSK9 inhibitors-associated AKI compared across age groups and genders.

Drug		Patients total	NO. AKI	Proportion of AKI (%)	ROR (95% CI)	*p*-Value
evolocumab	males	48,973	216	0.44	1.40 (1.54–1.69)	<0.001
	females	65,195	206	0.32	1 (reference)	
	25–65	33,388	117	0.35	0.80 (0.64–1.00)	0.05
	≥65	50,908	223	0.44	1 (reference)	
alirocumab	males	8,436	49	0.58	0.84 (0.59–1.20)	0.33
	females	12,154	84	0.69	1 (reference)	
	25–65	5,369	48	0.89	1.42 (0.97–2.09)	0.07
	≥65	9,365	59	0.63	1 (reference)	

Because the number of cases between 0–25 years old was small, they were not included in the comparison. Abbreviations: CI, confidence interval; ROR, reporting odds ratio.

## 4 Discussion

Despite numerous clinical studies confirming the lipid-lowering effects of PCSK9 inhibitors in CKD patients and recent research indicating their potential to improve CKD, their protective role in AKI remains unclear. Most cases of AKI are transient and challenging to intervene in practical clinical trials. Additionally, case reports have suggested that PCSK9 inhibitors may cause acute renal tubular injury. Therefore, investigating the correlation between PCSK9 inhibitors and AKI in a large-scale pharmacovigilance database study is crucial.

This study, based on the FAERS database, analyzed the association between PCSK9 inhibitors evolocumab and alirocumab and AKI. The results revealed a protective effect of PCSK9 inhibitors against AKI. Moreover, the study identified the main characteristics of AKI cases related to PCSK9 inhibitors and explored the impact of PCSK9 inhibitors in combination with common nephrotoxic drugs on AKI. To our knowledge, this is the largest real-world study investigating the risk of AKI associated with PCSK9 inhibitors.

While some case reports have linked PCSK9 inhibitors to AKI, this retrospective large-scale pharmacovigilance analysis showed a reduced risk of AKI in patients using PCSK9 inhibitors compared to those who did not. Both target drugs, evolocumab and elirocumab, exhibited similar effects with ROR of 0.13 (95% CI 0.12–0.14) and 0.26 (95% CI 0.23–0.30), respectively. Subgroup analysis also reached consistent conclusions. Studies have indicated that PCSK9 inhibitors are associated with anti-inflammatory (Giunzioni et al., 2016; Liu and Frostegård, 2018), autophagic (Ding et al., 2018; Huang et al., 2022), and oxidative stress responses (Huang et al., 2022), which are independent of low-density lipoprotein reduction. Recent research has revealed that these effects are mediated by SIRT3 (D'Onofrio et al., 2023), a highly expressed mitochondrial deacetylase, which plays a vital role in preventing AKI by regulating energy metabolism, inhibiting oxidative stress, suppressing inflammation, improving apoptosis, inhibiting early fibrosis, and maintaining mitochondrial homeostasis (Yuan et al., 2023). Activation of TFEB-mediated autophagy can also alleviate mitochondrial dysfunction in cisplatin-induced AKI (Zhu et al., 2020).

A large multicenter cross-sectional study summarized common nephrotoxic drugs in medical institutions (Liu et al., 2021). We conducted a disproportionate analysis of these drugs in combination with PCSK9 inhibitors and found that PCSK9 inhibitors can reduce the occurrence of AKI caused by common nephrotoxic drugs such as furosemide, pantoprazole, omeprazole, and esomeprazole. Therefore, considering PCSK9 inhibitors in patients with a high risk of AKI or those using nephrotoxic drugs seems wise. Additionally, existing real-world data studies have also shown the protective effect of PCSK9 inhibitors on AKI caused by medications. For example, the relative risk (RR) between the use of evolocumab and the occurrence of contrast-induced acute kidney injury (CI-AKI) was 0.34 (95% CI 0.17–0.66,*p* < 0.01) (Ma et al., 2022). It is worth mentioning that the common nephrotoxic drugs we selected may not be applicable to all countries and regions, although these drugs were derived from a nationwide cross-sectional survey that included 23 academic hospitals in 17 provinces in China. For example, cephalosporins and carbopenems may be uncommon as common nephrotoxic drugs. However, a pharmacovigilance study of FAERS found AKI ROR of cephalosporins is 6.07 (5.23–7.05) (Patek et al., 2020). Other studies believe that ceftriaxone calcium crystals induce AKI by NLRP3-mediated inflammation and oxidative stress injury (Yifan et al., 2020). Further investigation is warranted to accurately determine the nephrotoxicity of carbapenems. Nevertheless, current evidence suggests that, when co-administered with vancomycin, carbapenems present a reduced risk of AKI compared to piperacillin-tazobactam (Rutter and Burgess, 2018; Chen et al., 2023). However, risk of AKI after piperacillin-tazobactam is comparable to meropenem without concurrent use of vancomycin (Su et al., 2023).

We conducted a risk analysis of PCSK9 inhibitors-related AKI in different genders and age groups. We found that evolocumab is more likely to induce AKI in males (ROR = 1.40, 95% CI = 1.54–1.69), while alirocumab showed the opposite trend (ROR = 0.84, 95% CI = 0.59–1.20), although the latter did not reach statistical significance. Due to limited case numbers for patients under 25 years old, we only compared the reporting differences related to PCSK9 inhibitor-induced AKI between the age groups of 25–65 and over 65. We found a slightly lower risk of AKI in the 25–65 age group with evolocumab use compared to those over 65, and a higher risk with alirocumab use in patients aged 25–65, although these differences were not statistically significant. We observed that males might be a risk factor for evolocumab-related AKI, consistent with previous research results (Siew et al., 2016). However, in another study (Hilmi et al., 2015) on risk prediction models for AKI in liver transplant patients, females were identified as a risk factor (OR = 1.8, 95% CI 1.18–2.88). In fact, studies indicate that alirocumab exhibits a more pronounced effect in reducing LDL-C levels in males compared to females (Vallejo-Vaz et al., 2018; Paquette et al., 2023). Upon performing an analysis based on each 50% reduction in LDL-C levels, there was observed a 24% lower risk of major adverse cardiac events (MACE) in females (*p* = 0.1094), and a 29% lower risk in males (*p* = 0.0125), respectively (Vallejo-Vaz et al., 2018). Consequently, it can be hypothesized that males may derive greater benefit from alirocumab therapy. Further large-scale clinical trials are necessary to substantiate this speculation. Since this study is based on publicly available spontaneously reported drug surveillance databases, the reporting of patient gender is not mandatory and may lead to missing data, potentially introducing bias to the study results. Additionally, the sample size in this study was limited, and the characteristics of the study subjects were not entirely consistent; hence, caution is needed when extrapolating the conclusions.

Our research results showed an increasing trend in PCSK9 inhibitors-related AKI reports since the drug’s market launch, reaching its peak in 2018, followed by a gradual decline, especially for evolocumab. Evolocumab-related AKI cases were more common in people over 65 years old. Unfortunately, adverse event reporting in the FAERS database is spontaneous and influenced by various factors, such as the duration of drug marketing, media attention, types of adverse reactions, drug categories, indications, and related regulatory policies. Moreover, we do not have access to all clinical information related to the reported AKI cases, including gender, age, underlying diseases, concomitant medications, surgical procedures, and other AKI risk factors, which may confound the results. Drug surveillance studies based on the FAERS database cannot establish a causal relationship or determine the incidence rate of PCSK9 inhibitor-related AKI. They can only provide preliminary evidence of the potential correlation between the drug and adverse events. Regarding the selection of target populations, our study included a broad population, which might introduce bias into the results.

## 5 Conclusion

This study, based on the FAERS database, identified signals related to AKI associated with two PCSK9 inhibitors, Evolocumab and Alirocumab, revealing the protective effect of PCSK9 inhibitors against AKI. Furthermore, when used in combination with common nephrotoxic drugs, these inhibitors can reduce the risk of AKI caused by these medications. This provides clinicians with more comprehensive grounds and reasons for selecting PCSK9 inhibitors, especially for patients with a higher risk of AKI or those concurrently using nephrotoxic drugs due to hyperlipidemia. However, further large-scale randomized controlled trials are still necessary to validate these findings.

## Data Availability

Publicly available datasets were analyzed in this study. This data can be found here: https://research.cchmc.org/aers/home.
