# Supplementary material for: Association between PCSK9 inhibitors and acute kidney injury: a pharmacovigilance study
Source: Front Pharmacol. 2024 Aug 1;15:1353848. doi: 10.3389/fphar.2024.1353848 (PMC11324468; doi:10.3389/fphar.2024.1353848)
Supplement: Supplementary file 1 [file DataSheet1.pdf]

PTs selected for this study

"acute kidney injury" "acute phosphate nephropathy" "anuria" "azotaemia" "continuous haemodiafiltration" "dialysis" "foetal renal impairment" "haemodialysis" "haemofiltration" "neonatal anuria" "nephropathy toxic" "oliguria" "peritoneal dialysis" "prerenal failure" "renal failure" "renal failure neonatal" "renal impairment" "renal impairment neonatal" "subacute kidney injury" "albuminuria" "blood creatinine abnormal" "blood creatinine increased" "blood urea abnormal" "blood urea increased" "blood urea nitrogen/creatinine ratio increased" "creatinine renal clearance abnormal" "creatinine renal clearance decreased" "creatinine urine abnormal" "creatinine urine decreased" "crystal nephropathy" "fractional excretion of sodium" "glomerular filtration rate abnormal" "glomerular filtration rate decreased" "hypercreatininaemia" "hyponatriuria" "intradialytic parenteral nutrition" "kidney injury molecule-1" "nephritis" "oedema due to renal disease" "protein urine present" "proteinuria" "renal function test abnormal" "renal transplant" "renal tubular disorder" "renal tubular dysfunction" "renal tubular injury" "renal tubular necrosis" "tubulointerstitial nephritis" "urea renal clearance decreased" "urine output decreased".

Supplementary Table 1 Method for calculating the ROR based on a 2x2 contingency table

	target AE	other Aes	total
target drugs	a	b	a+b
other drugs	c	d	c+d

ROR: Reporting Odds Ratio; AE: adverse events;

a: Number of cases of the target adverse event associated with the target drug.

b: Number of cases of other adverse events associated with the target drug.

c: Number of cases of the target adverse event associated with other drugs.

d: Number of cases of other adverse events associated with other drugs.

Supplementary Table 2 PCSK9 inhibitor-related AKI case outcomes in FAERS database

drug	died	disabled	hospitalized	life threatening	other outcomes	required intervention	congenital anomaly
evolocumab	17	12	132	24	300	0	0
alirocumab	9	6	56	6	118	1	0

Note: since a patient's outcome may have multiple descriptions, the sum of these different outcomes of evolocumab and alirocumab is greater than 444 and 172.
